# A Concise Atlas of Thyroid Cancer Next-Generation Sequencing Panel ThyroSeq v.2

**DOI:** 10.4274/2017.26.suppl.12

**Published:** 2017-01-09

**Authors:** Jorge Alsina, Raul Alsina, Seza Gulec

**Affiliations:** 1 Florida International University Herbert Wertheim College of Medicine, Miami, USA; 2 Florida International University Herbert Wertheim College of Medicine, Departments of Surgery and Nuclear Medicine, Miami, USA

**Keywords:** thyroid cancer, next generation sequencing, ThyroSeq

## Abstract

The next-generation sequencing technology allows high out-put genomic analysis. An innovative assay in thyroid cancer, ThyroSeq® was developed for targeted mutation detection by next generation sequencing technology in fine needle aspiration and tissue samples. ThyroSeq v.2 next generation sequencing panel offers simultaneous sequencing and detection in >1000 hotspots of 14 thyroid cancer-related genes and for 42 types of gene fusions known to occur in thyroid cancer. ThyroSeq is being increasingly used to further narrow the indeterminate category defined by cytology for thyroid nodules. From a surgical perspective, genomic profiling also provides prognostic and predictive information and closely relates to determination of surgical strategy. Both the genomic analysis technology and the informatics for the cancer genome data base are rapidly developing. In this paper, we have gathered existing information on the thyroid cancer-related genes involved in the initiation and progression of thyroid cancer. Our goal is to assemble a glossary for the current ThyroSeq genomic panel that can help elucidate the role genomics play in thyroid cancer oncogenesis.

## Abstract (tr)

Tiroid nodüllerinde kanser teşhisi ve tiroid kanserlerinin prognostik değerlendirilmesi için geliştirilmiş en güncel yöntem DNA dizinleme teknolojisine dayalı ThyroSeq® testidir. Bu test ince iğne aspirasyon materyalinde genetik profilleme yaparak tiroid kanserinin tetiklenmesinde ve gelişmesinde rol alan mutasyon ve genetik değişiklikleri tespit etmektedir. Bu makalede bu test ile tespit edilebilen genetik değişikliklere konu olan genlerin yapı ve fonksiyonlarını kısaca tanımlıyoruz. Tiroid kanseri onkogenezine bir ölçüde ışık tutan bu test için sözlük görevi tutan bir makale sunulmaktadır.

## INTRODUCTION

Thyroid nodules are prevalent in the general population. Most thyroid nodules are benign and the clinical challenge is to accurately identify those nodules that are malignant and need to be surgically removed (1). Moreover, the extent of initial surgical treatment requires better understanding of particular tumor biology beyond conventional definitions. Molecular pathology is the new paradigm in cancer diagnosis and prognostication. Thyroid cancer develops and progresses through accumulation of genetic alterations, which does serve as important diagnostic, prognostic, and predictive biological markers (2). Next-generation sequencing technology allows high out-put genomic analysis. An innovative assay in thyroid cancer - ThyroSeq® - was developed for targeted mutation detection by next generation sequencing technology in fine needle aspiration and tissue samples. ThyroSeq v.2 next generation sequencing panel offers simultaneous sequencing and detection in >1000 hotspots of 14 thyroid cancer-related genes and for 42 types of gene fusions known to occur in thyroid cancer (3). ThyroSeq is being increasingly used to further narrow the indeterminate category defined by cytology for thyroid nodules. From a surgical perspective, understandably this provides prognostic and predictive information as it relates to determination of surgical strategy. Both the genomic analysis technology and the data collection for the cancer genome atlas are rapidly developing.

This paper reviews basic genomic information on 23 thyroid cancer-related genes involved in thyroid cancer. We have detailed information in regards to the location, and function of these genes in normal thyroid cells. We also report gathered information as to the consequences mutations to these 23 genes can have on thyroid cancer initiation and progression. Our goal is to provide a detailed glossary for ThyroSeq mutation panel.

## Molecular Markers of ThyroSeq Next-Generation Sequencing Panel

### B-RAF

**Figure 1:**
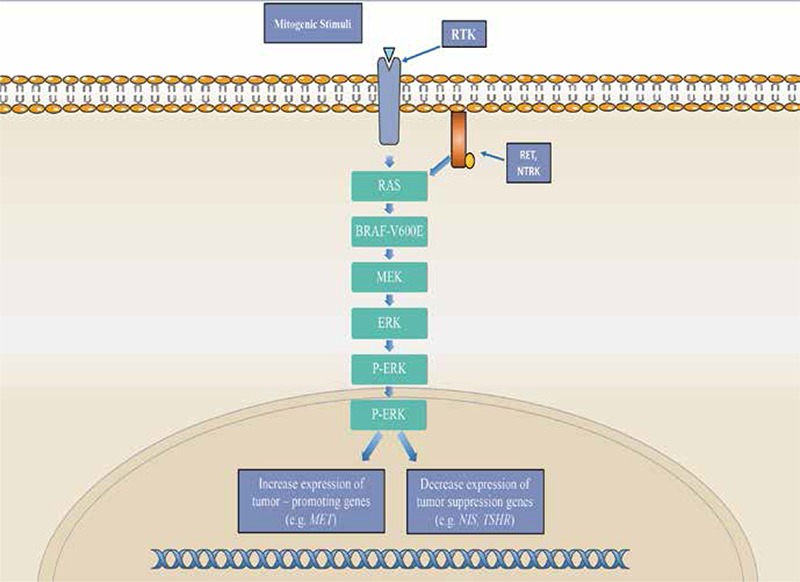
MAPK/ERK pathway *This pathway begins with a mitogenic stimulus binding to a receptor tyrosine kinase, activating it. This triggers a cascade of protein activation, beginning with RAS and culminating with ERK. Once ERK is phosphorylated, it enters the nucleus and influences transcription, increasing expression of tumor-promoting genes and decreasing expression of tumor-suppressing genes. MAPK signaling can also be stimulated by genetic alterations in proteins RET and NTRK

The B-RAF gene, located on chromosome 7q34, encodes B-RAF serine-threonine kinase, which functions as an intracellular effector of the RAS/MAPK signaling cascade (Figure 1). This is one of the three isoforms of the RAF serine-threonine kinase and the predominant isoform found in thyroid follicular cells. In wild-type forms of this gene, the B-RAF protein is activated through binding of a RAS-GTP protein complex with the B-RAF’s RAS binding domain along with simultaneous conformational changes in the protein. Once activated, the B-RAF protein phosphorylates the next protein in the signal cascade-MEK and ERK. The protein’s function contributes to the RAS/MAPK pathway’s role in cell proliferation, migration, and differentiation (4,5).

The most common B-RAF mutation found in thyroid carcinomas is a point mutation at residue 600 involving a substitution from valine to glutamate (V600E). This mutation results in the constitutive activation of the B-RAF protein and subsequently the RAS/MAPK pathway. The activation of the B-RAF protein seems to be caused by a disruption of the hydrophobic interactions between its activation loop and the ATP binding site. In wild-type B-RAF, these hydrophobic interactions help maintain the protein in an inactive conformation. When disrupted, B-RAF remains in an active, catalytic conformation. This results in the constitutive phosphorylation of its downstream targets (4).

The B-RAF V600E point mutation is most prevalent in papillary thyroid carcinomas (PTC)-the most common form of well differentiated thyroid carcinoma-found in 45% of PTC cases. Though it is rare in follicular variants of thyroid carcinoma, B-RAF is an ideal genetic marker for use in a thyroid cancer sequencing panel. It is found in all forms of thyroid carcinoma and seems to play a very important role early in tumorigenesis as a driver mutation (4,5,6).

### RAS

**Figure 2:**
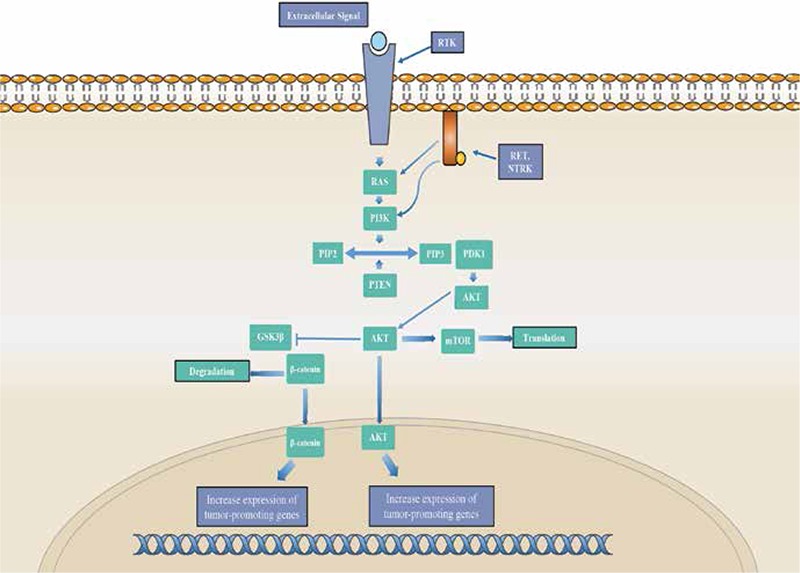
PI3K/AKT pathway *The pathway begins with an extracellular stimuli activating a receptor tyrosine kinase, triggering the activation of RAS followed by PI3K. Activated PI3K catalyzes the conversion of phosphatidylinositol (4,5)-bisphosphate (PIP2) to phosphatidylinositol (3,4,5)-trisphosphate (PIP3), a step that is regulated by PTEN. PIP3 activates the 3-phosphoinositide-dependent protein kinase 1, which targets and phosphorylates AKT. Phosphorylated AKT then enters the nucleus, where it increases the expression of tumor-promoting genes. Activated AKT also produces changes in the cytoplasm by activating mTOR and thus promoting translation. AKT can also phosphorylate glycogen synthase kinase 3-β (GSK3-β), inactivating the protein. GSK3-β inhibition results in a concomitant stimulation of β-catenin, allowing it to enter the nucleus and increase expression of tumor-promoting genes

The RAS genes consist of a family of highly homologous isoforms: K-RAS, N-RAS, and H-RAS. Located on chromosomes 12p12.1, 11p5.5, and 1p13.1 respectively, all three genes encode G-proteins located on the inner surface of the cell membrane. These proteins help convey signals from receptor tyrosine kinases (RTKs) to the pathways RAS regulates: RAS/MAPK and PI3K/AKT signaling cascades (Figure 2). Once bound to GTP, the RAS proteins proceed to activate the RAS/MAPK pathway. The RAS-GTP complex quickly becomes inactive as a result of the protein’s innate GTPase activity (7).

Point mutations in the RAS gene are the most common mutations. Mutations in codons 12 and 13 lead to an increased affinity for GTP. Mutations in codon 61 lead to inactivation of the RAS protein’s innate GTPase function. Overall, these mutations result in the constitutive activation of the RAS protein and thus the activation of the downstream signaling pathways it regulates, a critical step in thyroid tumorigenesis. Thyroid carcinoma has been associated with mutations in all three isoforms of the RAS genes, though it seems the more prevalent mutated isoform is that of N-RAS. However, the actual pattern of isoform frequencies in thyroid carcinoma remains unclear (4,7).

RAS mutations occur with variable frequency in all types of thyroid follicular-derived tumors. RAS point mutations are most common in follicular thyroid carcinoma (FTC), occurring in 40-50% of tumors, as well as in poorly differentiated and anaplastic thyroid carcinoma (PDC and ATC). It is more infrequent in PTCs, occurring in about 10% of tumors. The prevalence of RAS mutations in thyroid carcinoma make it a viable genetic marker as well as a useful prognostic tool, given that, studies suggest it may increase the potential for malignant transformation and tumor progression (7).

### CTNNB1

The CTNNB1 gene, located on chromosome 3p21, encodes a cytoplasmic protein known as β-catenin. This protein plays several important roles in the cell. It is involved in E-cadherin mediated cell to cell adhesion, found primarily in adherens junctions. It is also an intermediate in the Wnt signaling pathway. Once activated by proteins upstream in the pathway, β-catenin is able to accumulate in the cytoplasm and eventually is translocated into the nucleus. In the nucleus, the protein works with T-cell factor/lymphoid enhancing factor (TCF/LEF) to modulate gene expression (8,9).

**Figure 3:**
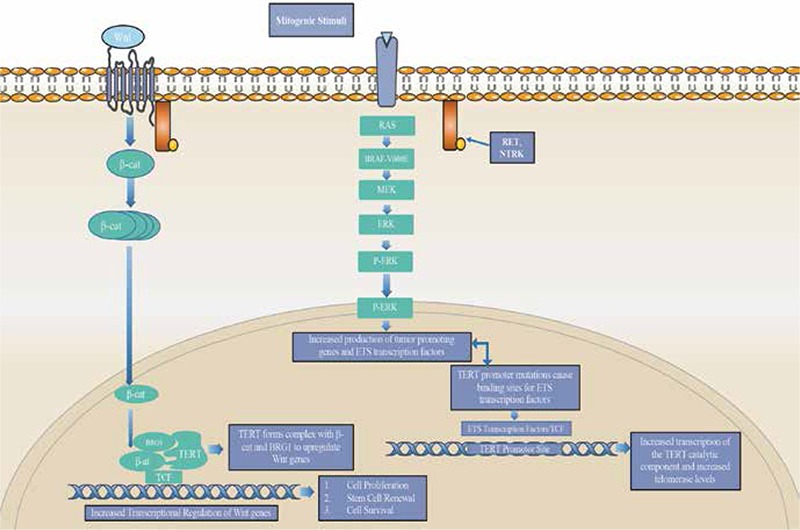
CTNNB1/TERT signaling *Dysregulation of telomerase is an almost universal feature of cancer, either through genetic amplification of the gene’s own locus or through crosstalk activity with other signaling pathways. In this pathway mutations to the promoter site of TERT (C228T and C250T) increase the transcription of TERT through the creation of consensus binding sites for ETS transcription factors. Constitutive action of the MAPK pathway due to B-RAF or RAS mutations leads to upregulation of TERT from ETS transcription binding activity. TERT also possess a role as a transcriptional modulator of the Wnt-β-catenin signaling pathway. Wnt stimulation at the plasma membrane leads to increased levels of β-catenin in the cytoplasm, and translocation to the nucleus. In the nucleus β-catenin forms a complex with TERT and the Wnt transcription factor BRG1. Once formed this complex binds to the promoters of Wnt target genes regulating the expression of these oncogenes

When β-catenin is not activated, free cytoplasmic levels are kept low through ubiquitin-proteasome degradation (Figure 3). However, point mutations in exon 3 of CTNNB1 confer added stability to the protein and thus inhibit the degradation of β-catenin. This results in the accumulation and localization of β-catenin in the cytoplasm and nucleus of the cell and the constitutive activation of its targeted genes (ex: Cyclin D1, C-MYC, C-JUN) (8,10,11).

Mutations in CTNNB1 are most commonly found in PDC and ATC but not in well differentiated forms. It is typically described as a late event in thyroid cancer progression, but dysregulation of β-catenin has been shown to contribute to tumor proliferation (10,11).

**Figure 4:**
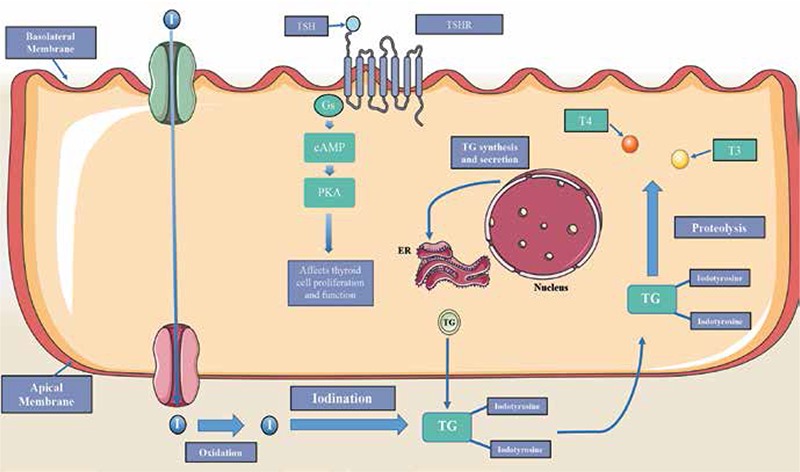
Thyroid hormone signaling *Follicular cells contain thyroid stimulating hormone receptors (TSHR). Once bound to the hormone TSH, this G-protein coupled receptor generates the second messenger cyclic AMP. This second messenger activates protein kinases that trigger signal cascades responsible for thyroid cell proliferation and function. TSH stimulation of TSHR also leads to iodide transport into the cell via the sodium iodide symporter. This iodide is oxidized and then bound to tyrosine residues on thyroglobulin (TG), a process catalyzed by thyroperoxidase. This primed form of TG can then be cleaved through proteolysis in the cell to generate the thyroid hormones T3 and T4

CTNNB1 can serve as a useful marker for PDC and ATC. Its involvement in the Wnt pathway contributes to its viability as a marker, given this pathway is constitutively active in 50% of PDC and ATC and it is involved in the dedifferentiation process of thyroid carcinoma (Figure 4) (10). This gene is thus a useful diagnostic marker as well as prognostic tool to determine the progression of the cancer and its propensity to spread.

### NTRK1

The NTRK1 gene, which is located on chromosome 1q21-22, encodes the neurotrophic RTK-1, also known as TrkA. It is a high affinity receptor for nerve growth factor-β (NFG-β) that provides instructions for the growth and development of nerve cells. It also induces the proliferation of a number of cell types, such as lymphocytes and keratinocytes. Once it is bound to NFG-β, NTRK1 dimerizes and autophosphorylates its five tyrosine residues, which then act as binding sites for several target proteins such as RAS and PI3K. By activating these proteins, NTRK1 subsequently activates the RAS/MAPK and PI3K/AKT signaling cascades, thus mediating NGF effects on the proliferation and differentiation of cells (12,13).

Mutations in the NTRK1 gene are frequently found in PTC. These mutations involve chromosomal rearrangements between NTRK1 and at least three other identified genes: TPM3, TPR, and TFG. The chimeric oncogenes that are formed (TRK oncogenes) encode constitutively active RTKs. It remains active due to the fact that its kinase domain and the tyrosine binding sites are preserved in the chimeric oncogene. The only portion that is lost is a portion of its extracellular domain. It seems that the extracellular domain may contain important regulatory elements. This provides constant activation of the protein signals for cell proliferation, contributing to PTC (12,13).

About 60% of PTC carry chimeric oncogenes created through chromosomal rearrangement. Though RET rearrangement and not NTRK1 is the most prevalent chimeric oncogene detected in PTC, thyroid epithelium seems to have a propensity for chromosomal rearrangements. That fact, along with NTRK1 association with pathways implicated in thyroid carcinoma (RAS/MAPK and PI3K/AKT) make this gene a viable marker for diagnostic and prognostic purposes. Nevertheless, further investigation is necessary to better understand its role in thyroid carcinogenesis (13,14).

### NTRK3

Another member of the neurotrophic tyrosine receptor kinase family, NTRK3 encodes a RTK with a high affinity for its ligand neurotrophin-3. The gene is located on chromosome 15q25. Once bound, NTRK3 autophosphorylates and proceeds to phosphorylate target proteins primarily involved in the RAS/MAPK and PI3K/AKT signaling pathways. Primarily expressed in the central nervous system, NTRK3 is involved in cell proliferation and differentiation as well as neuronal cell processes (14,15).

The mutation of NTRK3 observed in thyroid carcinomas is a chromosomal rearrangement involving ETV6, a gene encoding a transcription factor from the ETS transcription factor family. This particular oncogenic fusion involves the fusion of exons 1-4, the SAM domain, of ETV6 and exons 12-18, the tyrosine kinase domain, of NTRK3. This results in the constitutive activation of the RTK. It is this constant activation that contributes to thyroid carcinogenesis (15,16).

Chromosomal rearrangements of the NTRK genes are uncommon in sporadic PTC, only occurring in about 2% of cases. However, there is higher prevalence of these chimeric oncogenes in pediatric and radiation-related PTC, with the majority of ETV6-NTRK3 fusions demonstrating the follicular variant of PTC. Mutations resulting in RAS/MAPK activation in radiation-related PTC tend to differ from those found in sporadic PTC as there tends to be more chromosomal rearrangements than point mutations (15,16).

### RET

The RET gene is a proto-oncogene that is highly expressed in parafollicular C-cells. Located on chromosome 10q11.2, this gene encodes a RTK that is involved in the RAS/MAPK signaling pathway. The RET protein responds to signals from the cell’s environment and then proceeds to activate itself through autophosphorylation. Once activated, the RET protein elicits changes within the cell often involving cell proliferation and development (4,17).

Chromosomal rearrangements involving RET represents one of the most common causes of PTC. At least 11 rearrangement variants have been isolated but the most common translocations are RET/PTC1 and RET/PTC3. The aforementioned rearrangements are intrachromosomal paracentric inversions and account for the vast majority of variants found in PTC. These rearrangements result in a constitutively active RET protein, leading to activation of the RAS/MAPK pathway and driving thyroid carcinogenesis (17,18).

The RET/PTC1 rearrangement involves interaction between RET and the H4 gene. This particular rearrangement is the dominant type in sporadic PTCs. The RET/PTC3 rearrangement involves interaction between RET and the ELE1 gene. This particular rearrangement is the dominant type in radiation-related PTCs, which tend to be more aggressive and exhibit a higher frequency of chromosomal rearrangements (4,17).

### AKT1

AKT1 codes for the AKT1 kinase, one member of a family of homologous isoforms. Located on chromosome 14q32.322, AKT1 kinase helps regulate cell growth and survival as well as the process of apoptosis, mainly through interaction with signaling pathways like PI3K/AKT. The protein contains a pleckstrin homology domain, making it a target for phospholipids generated by PI3K activity. Once activated through phosphorylation, AKT1 binds to chaperone proteins and proceeds to phosphorylate its downstream effectors. Through this pathway, activated AKT1 is able to inhibit apoptosis and induce cell growth in thyroid cells (19).

Activation of AKT1 is known to promote thyroid tumorigenesis in Cowden’s syndrome (CS). It is the overexpression and overactivation of AKT1, rather than mutation of the AKT1 gene, that contributes to the pathogenesis of sporadic thyroid cancers, particularly in follicular thyroid cancers (FTC). In FTCs, activation of AKT1 kinase and the localization of the protein in the nucleus promotes the PI3K/AKT pathway, contributing to tumor invasion and metastasis. It seems that overexpression of AKT1 is capable of promoting cell proliferation, sensitization to TSH, and inhibiting apoptosis, but it is not sufficient to transform thyroid cells by itself (19,20).

This alteration in AKT1 occurs in about 15% of metastatic thyroid cancers, while also occurring in small fractions of ATCs and FTCs. Studies have shown that there is an association between AKT1 activation and tumor aggressiveness. This association with metastasis in thyroid carcinoma was found in both PTC and FTC. However, the aggressiveness seen is likely attributed to a combination of genetic alterations rather than solely on dysregulation of AKT1 activity (19,20,21).

### TERT

The TERT gene, located on chromosome 5p15.33, encodes the protein telomerase reverse transcriptase. It is the catalytic subunit of the telomerase enzyme and determines telomerase activity. The TERT subunit associates with a RNA component TERC as well as additional components to form the telomerase enzyme. Telomerase serves an important role in synthesizing and maintaining telomeres, which helps prolong the life of cells in the body. When telomeres become too short, apoptosis is triggered in the cell. As a result, telomerase activity is highly regulated and expressed at very low levels in most tissues in the body (22).

Dysregulation of telomerase is an almost universal feature of cancer, with over 90% exhibiting overexpression of the enzyme. This imparts cancer cells with an infinite capability to divide. The up-regulation of TERT that is seen could be the result of epigenetic deregulation or genetic amplification of the gene’s locus (Figure 3). Other factors contributing to the dysregulation of TERT expression involve changes in transcription factors that target the TERT gene. Such factors include TP53 and the ETS transcription factors. Both are disrupted in thyroid carcinoma as TP53 is often mutated and ETS transcription factors are stimulated by several oncogenes including RAS. These associations indicate a relationship between an up-regulation of TERT expression and the transformation of thyroid cells. One recently discovered mechanism has been mutations in the TERT promoter, the most important regulatory element of telomerase activity (22,23).

These promoter mutations, C228T and C250T, seem to increase the transcription of TERT, likely through the creation of consensus binding sites for ETS transcription factors. Promoter mutation C228T is the most common one found in thyroid carcinoma with no overlaps reported in the mutations. There seems to be a higher prevalence of TERT promoter mutations in more advanced forms of thyroid carcinoma like ATC. Studies have shown a strong association between TERT promoter mutations and B-RAF or RAS mutations as well. The evidence uncovered so far shows that mutations in the TERT promoter seems to represent a late stage genetic event in tumorigenesis as well as an indicator of aggressiveness given its prevalence in ATC (24,25).

### GNAS

The GNAS gene provides instructions for the stimulatory α-subunit of heterotrimeric G protein complexes. The gene is located on chromosome 20q13.3. This subunit stimulates the activity of adenylate cyclase and controls the production of several hormones that help regulate the activity of the endocrine glands such as the thyroid gland. Mutations in GNAS are often detected at codons 201 and 227. These mutations result in the constitutive activation by impairing GTP hydrolysis. This in turn results in the constitutive activation of the cyclic AMP (cAMP) cascade. The increase in cAMP levels results in thyroid cell proliferation and increased thyroid hormone production. Despite this disruption in function, these mutations are almost never malignant (20,26).

Activating mutations of GNAS has been shown to overactivate thyroid stimulating hormone receptor (TSHR) signaling. This often leads to benign, hyperfunctional follicular thyroid adenomas (FTAs). Mutation of GNAS is also the main cause for autonomously functioning thyroid nodules. Studies show that mutations in GNAS resulting in overactivation of the G protein alpha subunit leads to a hyperfunctioning thyroid. Serving as a potential marker for benign thyroid nodules, GNAS does not seem to serve as a driver of thyroid carcinogenesis. However, it seems likely that this disruption in thyroid function in conjunction with other genetic alterations plays a role in thyroid carcinoma (26).

### PIK3CA

PIK3CA, phosphatidylinositol-4,5-bisphosphate 3-kinase catalytic subunit alpha, is a gene located on chromosome 3q26.3. It encodes the p110 alpha subunit of the PI3K kinase. This particular subunit is the catalytic subunit and is responsible for the kinase function of the protein. PI3K plays a very important role in the PI3K/AKT signaling pathway, one of the major pathways implicated in thyroid carcinoma. As part of this signaling pathway, PI3K helps regulate cell proliferation, motility, adhesion, and cell cycle progression. The pathway begins with signals transmitted from the environment to the inside of the cell through RTKs. These RTKs then engage PI3K, recruiting the protein to the cell membrane and generating phosphatidylinositol-3,4,5-triphosphate (PIP3). PIP3 acts as a second messenger, recruiting protein kinases phosphoinositide-dependent protein kinase 1 (PDK1) and AKT to the membrane. Once activated via phosphorylation, these kinases target their downstream effectors, producing concomitant changes to cell growth and differentiation. This pathway is regulated by the PTEN lipid phosphatase, which dephosphorylates PIP3 and thus shuts off the PI3K/AKT pathway (27).

The PI3K pathway is frequently activated in thyroid carcinoma and studies have reported the overexpression of PIK3CA in FTC and ATC. The most common PIK3CA mutations observed with oncogenic effects tend to cluster around exons 9 and 20. These mainly somatic mutations result in the constitutive activation of PI3K. Consequently, this leads to overactivation of the PI3K/AKT pathway and thyroid tumorigenesis. Genetic alterations have been found in all differentiated stages of thyroid carcinoma. However, it seems to be the most frequent in ATC, occurring in about 24% of ATC cases. Given the occurrence of PIK3CA mutations in all stages of thyroid carcinoma, it supports the implication of the PI3K/AKT signaling pathway in both the initiation and progressive dedifferentiation of the disease (27,28,29).

### PTEN

PTEN, phosphatase and tensin homolog, encodes a phosphatase enzyme found in almost all tissues in the body. Located on chromosome 10q23.3, PTEN plays an important role in the PI3K/AKT pathway. Once activated via phosphorylation, PTEN works to terminate the signaling pathway through its phosphatase activity. It converts PIP3 into PIP2and shuts off the PI3K/AKT pathway and reduces the recruitment of kinases PDK1 and AKT. As a result, this protein helps regulate cell proliferation and differentiation (28,29).

A tumor suppressor gene, PTEN is found mutated frequently in thyroid tumors. Point mutations, deletions, and promoter methylation in PTEN have been reported in thyroid carcinoma, particularly undifferentiated ones like ATC. Overall, studies have shown that PTEN inactivation is a critical step in thyroid tumor progression. This is evident in the fact that PTEN is downregulated or absent in highly malignant thyroid carcinoma like ATC, though mutations have been found in more differentiated forms. PTEN downregulation has been reported in about 37% of FTCs. Ultimately, this inactivation results in the overactivation of the PI3K/AKT signaling pathway, resulting in rampant cell proliferation (28,29,30).

PTEN mutations tend to be most frequent in ATC, with an occurrence in about 15% of cases. Germline mutations of PTEN is also a leading cause of CS, a disorder that can result in benign and malignant tumors. Individuals with CS have a 10% risk of developing thyroid cancer. This association between CS and PTEN further supports its role in thyroid tumorigenesis (27,29,30,31).

### TSHR

The TSHR gene, located on chromosome 14q31, encodes the TSHR. This G protein-coupled receptor spans the membrane of follicular cells in the thyroid. Once activated by thyroid stimulating hormone (TSH), TSHR produces corresponding effects in the cell via second messengers like cAMP. This intracellular signaling plays an important role in thyroid cell proliferation and maintenance of thyroid function (Figure 4). Deregulation of TSHR seems to play an important role in thyroid carcinogenesis (20).

Studies have shown an association between high levels of TSH and the development of thyroid nodules. The overactivation of TSHR, whether through activating mutations in TSHR or GNAS, has also been shown to lead to hyperfunctional FTA. However, these tumors are rarely malignant, suggesting that TSHR could serve a dichotomous role - protecting against malignancy of thyroid tissue but also promoting carcinogenesis likely when activated by other oncogenic alterations. Mouse models have shown that TSHR is required for thyroid carcinogenesis. It is still uncertain whether this supports TSHR’s role in the initiation of thyroid carcinoma or it is simply due to the TSHR-dependent generation of thyroid cells from which carcinogenesis initiates (20,32,33).

Epigenetics has shown to play an integral part in the role of TSHR in thyroid carcinoma. Hypermethylation of the TSHR promoter has been frequently found in thyroid carcinoma, while it is unmethylated in normal and benign thyroid tumors. This epigenetic silencing is most prevalent in PTC, with a frequency of 34-59%. Epigenetic silencing via hypermethylation is also prevalent in more undifferentiated forms of thyroid carcinoma. Studies have also shown a strong association with B-RAF mutations in PTC. This relationship supports the theory that TSHR silencing is a secondary genetic event in thyroid carcinogenesis and not essential for the initiation of thyroid carcinoma (32,33).

The silencing of TSHR not only contributes to the development of thyroid carcinoma, but it desensitizes the thyroid tumors to radioiodine therapy through its interaction with sodium iodide symporter (NIS) in iodide uptake. The suppression of TSHR hinders the cancer cells’ ability to concentrate iodine. Though no silencing mutations have been found thus far, it seems that methylation of TSHR serves as a marker for thyroid malignancy (33).

### TG

The TG gene located on chromosome 8q24, encodes the prohormone TG. This protein, found in thyroid follicular cells, acts as a precursor for the thyroid hormones, serving as the matrix from which they are produced. It acts as a protein storehouse and when the thyroid hormones are needed, TG is altered and broken down to release the needed hormones. Tyrosine residues on the TG protein are iodinated, priming the protein for modification into the thyroid hormones T3 and T4 (Figure 4). The synthesis of TG is regulated by TSH, with TSH serving to stimulate production (34).

Mutations in TG have been reported in thyroid carcinoma. They tend to be more frequently found in differentiated thyroid carcinomas, like PTC. The majority of PTCs with a mutation in TG tend to also carry a mutation in one of the genes in the MAPK signaling pathway. Though rarely mutated, TG does seem to contribute to the malignancy of the cancer. As the cancer dedifferentiates, the expression of TG seems to decrease, with it becoming absent in ATC. These genetic changes do not seem to be sufficient to initiate thyroid carcinogenesis, but the hormonal imbalance produced seems to aid in its progression (34,35).

Changes in TG are more commonly associated with dyshormonogenesis, occurring with a frequency of 25%. Individuals with the disorder tend to develop goiters, often due to chronic stimulation by TSH. Studies have shown that individuals with these dyshormonogenic goiters and TG mutations have an increased risk of developing aggressive forms of thyroid carcinoma.

TG also serves as a tool for monitoring thyroid cancer recurrence and metastasis after treatment and thyroidectomy. Serum TG levels are carefully monitored and upon stimulation by TSH, if levels rise too high it serves as a strong indicator of metastases. Such an association helps support the role of TG dysregulation in the development of thyroid carcinoma (35,36,37,38).

### PAX8/PPARγ

A chromosomal rearrangement found in thyroid carcinoma is that between the genes PAX8 and PPARγ, PAX8 encodes a transcription factor that is essential for normal thyroid development. The PPARγ gene encodes a nuclear receptor part of the PPAR subfamily. This receptor forms heterodimers with retinoid X receptors and helps regulate the transcription of several genes, playing a major role in adipogenesis and lipid metabolism (3,39).

This rearrangement is the result of a translocation between chromosome regions 2q13 and 3p25. Highly expressed in thyroid follicular cells, it seems this fusion protein plays a role in thyroid tumorigenesis. The exact mechanism is still unknown. Some studies show that PAX8/PPARγ has a dominant negative effect on wild type PPARγ, which is believed to act as a tumor suppressor. The fusion protein contains the transcriptional regulatory domains of both proteins. As a result, it is capable of modulating their respective downstream pathways, usually targeting PAX8-responsive promoters (39,40).

PAX8/PPARγ is most frequently found in FTCs, with an occurrence rate of about 30%, and occurs rarely in the follicular variant of PTC. The presence of this rearrangement has no major impact on the prognosis of thyroid carcinoma. Though still much remains unknown about this chromosomal rearrangement, evidence points to its potential as an oncoprotein and genetic driver of thyroid carcinoma (4,39,40).

### IGF2BP3/IMP3

The IGF2BP3 gene is located on chromosome 7p11.2, and encodes for the insulin-like growth factor-2 (IGF-2) mRNA-binding protein 3. IGF2BP3 is a RNA-binding protein consisting of two RNA-recognition motifs in its N¬-terminal part and four KH homology domains in the C-terminal region. Its primary function is as a translational activator of the IGF-2 gene, while also functioning as a post-transcriptional regulator of cell proliferation, and differentiation during embryogenesis. It is important to note that IGF2BP3 is a member of the highly conserved IGF2BP family of mRNA-binding proteins along with IGF2BP1 and IGF2BP2. As a member of the IGF2BP family IGF2BP3 shares a high amount of similarity to the other three members at the amino acid level but differs in terms of its expression. IGF2BP3 is abundantly found in all tissues during embryonic stage, but it’s expression in normal mature tissue is confined to the placenta and reproductive tissue (41,42,43).

IGF2BP3 has been the member of the IGF2BP family to be most associated with various cancer types such as lung, ovarian, pancreatic, and colorectal. Its role in tumor progression has been suggested to be by promoting cell growth, proliferation and resistance to ionic radiation via an IGF-2 dependent manner. Recent studies have found IGF2BP3 to play a role in increasing the invasive potential of tumor cells in vitro (42,43).

Due to the high transcript levels of IGF2BP3 in neoplastic cells and near undetectable presence in most adult tissue, it’s been suggested as a potential biomarker. Through the use of immunoassays, its expression has been exclusively observed in malignant thyroid cancers of follicular origin, with a small portion of classical PTC, and was observed in 59% of cases for poorly differentiated thyroid carcinoma variants. However, through the use of reverse transcription polymerase chain reaction (RT-PCR) and qRT-PCR assay, it was demonstrated that IGF2BP3 was overexpressed in thyroid carcinoma tissue when compared to benign thyroid lesions. IGF2BP3’s overexpression in thyroid carcinoma, its near undetectable levels in adult tissue, and its potential to distinguish between benign and malignant tissue make it an excellent candidate for the thyroid cancer sequencing panel (41,44,45,46).

### KRT7/KRT20

The KRT7 and KRT20 genes both belong to the keratin gene family, which consists of 54 functional genes. The KRT7 gene can be found in type 2 cluster on chromosome 12, while the KRT20 gene is located in the type 1 cluster on chromosome 17. The protein products of these genes are subdivided into type 1 and type 2 based on their molecular weights. KRT7 and KRT 20 both encode for cytokeratin proteins that are intermediate filament forming proteins exclusively found in epithelial tissue. The filaments are formed from the heterodimer pairing of type 1 and type 2 cytokeratin proteins in equimolar amounts. The intermediate filaments created by cytokeratin proteins aid in forming the structural framework of epithelial cells, regulate cell size, growth, division, as well as protection against mechanical stressors (46,47).

Adenocarcinomas compromise the largest group of human epithelial malignancies and can occur in different organs. KRT7 and KRT20 along with the other members of the keratin gene family have been utilized as diagnostic markers to determine the origin tissue, due to the characteristic cell-type, differentiation and functional status-dependent expression pattern of cytokeratin in epithelial cells. In most adenocarcinomas, the expression levels of KRT7 and KRT20 are variable and thus are often screened for together (47).

Current literature on KRT7/KRT20 combined screening of thyroid neoplasms is scarce. Almost all thyroidal tumor’s expression levels for KRT7 and KRT20 have been found to be positive and negative, respectively. In fact, one study looked into the immunohistochemical profile of 43 different types of primary and metastatic thyroid neoplasms. They found that excluding anaplastic carcinomas, 79% of thyroid tumors and 94% of metastatic thyroid carcinoma cases reacted positively with the antibody. While the study found a positive result for KRT7, none of the thyroid tumors or their metastases reacted at all to the KRT20 antibody. A second retrospective study examined 153 thyroid carcinoma samples for KRT7/KRT20 immunohistochemically, and they found that all papillary carcinomas, follicular carcinomas, and medullary carcinomas were KRT7 positive and KRT20 negative. Due to the fact that KRT7 has been positively stained in almost all thyroid neoplasms while KRT20 has consistently been undetectable, KRT7 and KRT20 are excellent candidates for the thyroid cancer sequencing panel (48,49,50).

### TP53

**Figure 5:**
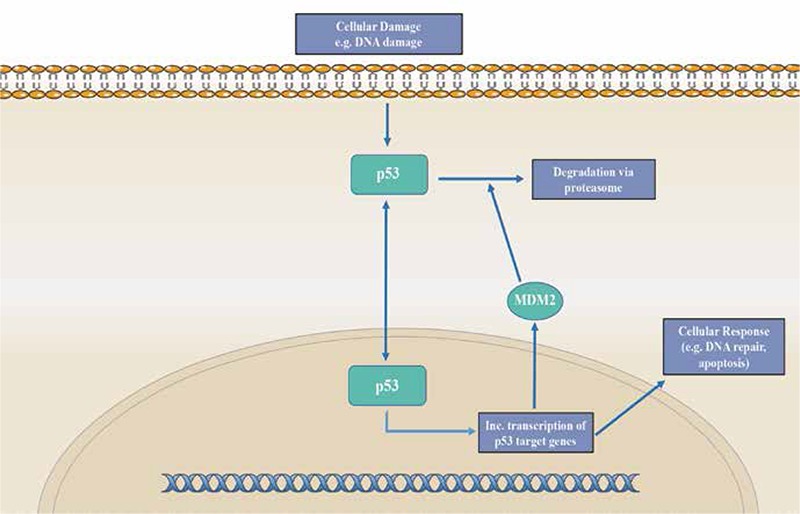
P53 pathway *Signals of cellular stress, such as DNA damage, triggers activation of p53. Once activated, this nuclear transcription factor travels to the nucleus and binds to DNA, increasing expression of target genes involved in DNA repair and triggering apoptosis. p53 activity also induces expression of MDM2 as well. MDM2 serves to regulate p53 activity, inhibiting transcription of p53 and promoting its degradation. Overall, this pathway functions as an auto-regulatory feedback loop

The gene TP53 encodes tumor protein p53, located on chromosome 17p13.1. This protein is a nuclear transcription factor that functions as a tumor suppressor. P53 contains three functional domains: a transactivation domain, a DNA binding domain, and an oligomerization domain. In the nucleus, it binds directly to DNA in order to help prevent mutated or damaged DNA from dividing. The protein either activates other genes to fix any damages or triggers cell cycle arrest and apoptosis (Figure 5). As a result, this protein plays a crucial role in regulating cell division and preventing tumor formation (51).

Any impairment in the function of TP53 can lead to destabilization of the genome and an accumulation of mutations. Most changes involve point mutations in exons 5-8, deletions, or inactivation through indirect mechanisms such as methylation. The now inactivated TP53 protein becomes unable to bind to DNA and thus is unable to repair damages that arise in the DNA. The protein, as well as damages to the DNA, just accumulates in the nucleus. These type of changes are usually restricted to poorly differentiated thyroid cancers like ATC, reportedly occurring in 60% of cases (8,51).

TP53 exhibits tremendous oncogenic potential, as it is mutated in about 50% of all human cancers. However, inactivating mutations of TP53 are found in only about 10% of thyroid carcinomas overall. In well differentiated thyroid carcinomas, TP53 mutations have a reported relevance ranging from 0 to 25%. Though it seems other mechanisms of TP53 inactivation play a role in thyroid carcinogenesis, as p53 protein is more commonly overexpressed with a frequency of 11-59% (51,52).

### CALCA

CALCA (CALCA-1) gene is a member of the calcitonin gene family consisting of four known genes, CALCA-1, CALCA-2, CALCA-3, and CALCA-4 all containing nucleotide sequence homologies. The CALCA gene located on chromosome 11 encodes for two separate peptide hormones. This gene consists of six exons in which exon 4 and 5 contain the sequences for the calcitonin and calcitonin gene related peptide (α-CGRP), respectively. Post-transcriptional processing of the CALCA gene in thyroid C cells and neural tissue results in tissue specific production of calcitonin and α-CGRP mRNAs. CT is a 32 amino-acid peptide hormone with tissue specific functional roles in the central nervous, skeletal, renal and gastrointestinal systems. However, CT’s major role is in calcium and phosphate metabolism. CT modulates calcium levels by binding with CT receptors to inhibit osteoclast motility. All of CT’s activities occur through its binding to the calcitonin receptor, which couples its binding to the activation of cAMP and PKC signaling pathways (53,54,55).

Due to the uncharacteristically high concentrations of calcitonin, serum CT is classically used as a diagnostic biomarker for MTC. Studies have demonstrated that preoperative serum CT levels can be strong predictors for tumor size and remission. Preoperative CT serum levels above 1000 pg/mL correspond to 25.0 mm tumor sizes, while levels below 100 pg/mL were associated with 3.0 mm tumor size. Distant metastases are the main cause of death in patients diagnosed with MTC. Metastasis is initially presented in 50% of MTC cases. Postoperative serum CT levels are an important procedure in screening for biochemical remission. CT doubling times have been found to be associated with progressive or stable disease in 80% of patients. In a study they found doubling times shorter than 25 months to have a 94% predictive value of progressive disease along with a predictive value of 86% for patients with a doubling time longer than 24 months (53,56,57,58,59).

CALCA detection studies and procedures involve detection of this peptide from blood serum. There are limitations to these assays due to needing stimulation tests, and possible intra-assay variation. Some research has found that detection of CALCA gene transcripts presented higher clinical sensitivity, specificity, and positive predictive values of 86.67, 97.06, and 92.86%, respectively. In addition to these promising results their readings mirrored measurements obtainable through a pentagastrin stimulation test. Due to its tissue specific production and role in preoperative and postoperative diagnosing of MTC, CALCA is a viable candidate to include in the thyroid cancer mutation panel (53,60).

### MET

MET, also known as c-MET, encodes a proto-oncogene that is located on chromosome 7q31. A member of the RTK family, the MET receptor elicits its effects by binding to its ligand, hepatocyte growth factor (HGF). Once bound to HGF, the MET receptor dimerizes and thus becomes activated and helps promote cell proliferation, survival, and motility. These effects are largely the result of activation of MAPK and PI3K pathways through MET signaling. This process is tightly regulated and any disruption can result in tumorigenesis (61).

In thyroid carcinoma, what is most commonly seen is a deregulation of MET, most commonly in PTC. This deregulation often involves overactivation or overexpression of MET, which has been shown to promote tumor growth. This activation can be the result of activating point mutations often located in the tyrosine kinase domain, or paracrine signaling due to increased sensitivity to HGF. The altered MET gene acts as a mitogenic factor and helps promote cell motility and invasion (61,62).

Expression levels of MET tend to be low and tightly regulated in normal thyroids. However, it is amplified in 75% of PTCs, as well as in many FTCs. This overexpression seems to be secondary to other driver mutations like RAS and B-RAF. Despite its secondary role, evidence points to increased MET signaling having a significant role in the pathogenesis of thyroid carcinoma (62,63,64).

### EIF1AX

This X-linked gene, located on Xp22.12, encodes the eukaryotic translation initiation factor 1A. This particular protein is required for the formation of the preinitiation complex (PIC). It does so by stabilizing the binding of the initiator tRNA (Met-tRNA) with the 40S ribosomal subunit and the translation initiation factor eIF2. This is an integral step in the translation of mRNA into proteins, as it is required for the binding of the PIC to the 5’ end of capped mRNA and finding the proper start codon (65,66).

Mutations in EIF1AX have been found in both PTC and ATC. The most common sites of mutation are the N-terminal domain and a C-terminal splice acceptor site between exons 5 and 6. The C-terminal mutations seem to be specific to thyroid carcinoma. In PTC, it is rare and occurs in a mutually exclusive manner, found in only 1-2% of tumors. But, it is more frequent in ATC, found in 10% of tumors. It seems that these mutations result in an overactive EIF1AX, as it has been shown to increase cell proliferation (65,66,67,68).

EIF1AX mutations tend to coexist with RAS mutations and other driver mutations of thyroid carcinoma like TP53. This co-occurrence tends to be found predominantly in poorly differentiated thyroid carcinomas like ATC. This strong association suggests that EIF1AX is not sufficient enough to transform thyroid carcinoma into a malignant form. Mutations in EIF1AX have also been associated with poor prognosis in PDCs. The mechanisms of EIF1AX mutation in thyroid carcinoma are still unclear, especially in terms of its association with RAS mutations (65,66,67,68).

### TTF1

Also known as NK2 homeobox 1, this gene encodes thyroid transcription factor 1. The TTF1 protein is expressed in the epithelial cells of the thyroid with a heterogeneous distribution. As a homeoprotein, it plays an important role in thyroid development, cell growth, and differentiation. Essential for thyroid organogenesis, TTF1 helps modulate thyroid function through its regulation of gene expression in the thyroid, activating the transcription of thyroid-specific genes like thyroglobulin (TG) and thyroperoxidase (TPO) (69,70).

The mechanisms by which TTF1 contributes to thyroid carcinoma still remains unclear. Though the gene is expressed in thyroid carcinoma, its level of expression becomes reduced as cell dedifferentiation progresses. This change in expression could be the result of epigenetic silencing, likely achieved through hypermethylation and histone H3 modification. As a result, ATC exhibits the lowest levels of TTF1. In thyroid carcinoma as a whole it seems likely that TTF1’s molecular functions, DNA binding, and transcription activation are probably disrupted. That could explain why most undifferentiated thyroid carcinomas have little to no TG or TPO proteins, both targets of TTF1 transcription factor activity (41,42,43).

Germline mutations in TTF1 have also been shown to play a role in thyroid carcinoma, specifically in PTC. These mutations seem to promote cell proliferation as well as activate signaling pathways like PI3K/AKT. The germline mutations observed in TTF1 have also been associated with multinodular goiter. This provides further evidence of a progression from a benign multinodular goiter to malignant thyroid carcinoma, such as PTC. Given the important role TTF1 plays in maintaining thyroid architecture and function, it is not surprising that any abnormalities in the gene and its product contribute to the development of thyroid carcinoma (70,71,72).

### PTH

The PTH gene encodes a member of the parathyroid family of proteins and is located on chromosome 11p15.3. This particular protein binds to the parathyroid hormone receptor and helps regulate blood calcium and phosphate levels in the body. Abnormal levels of PTH tend to be associated with parathyroid diseases, like hyperparathyroidism. However, there is evidence to suggest a possible link between PTH and the development of thyroid carcinoma (73).

Primary hyperparathyroidism (PHPT) is a disease that has been associated with thyroid carcinoma. PHPT results from an excessive secretion of PTH, which causes an elevation of calcium in the blood. The most common form of thyroid carcinoma associated with PHPT is a hereditary form of MTC known as multiple endocrine neoplasia type 2A (MEN2A). Carriers of the MEN2A gene with a mutation at codon 634 concomitantly suffer from PHTP with a frequency of 10-25%. Such an association provides evidence to support the role of PTH dysregulation in thyroid carcinoma (73,74).

Associations between non-medullary thyroid carcinoma and PHPT have also been reported. With rates ranging from 2-10%, thyroid malignancy seems to coexist with PHPT, albeit not frequently. A strong relationship has also been found between ionizing radiation and the simultaneous development of PHPT and thyroid carcinoma. PTH may also contribute to the development of thyroid carcinoma through chromosomal rearrangement with PAD1. This rearrangement results in an overexpression of cyclin D1 and thus an increase in cell proliferation. Though some changes to PTH may be the result of metastatic invasion of the parathyroid by thyroid carcinoma, the evidence available shows that the role of PTH in thyroid malignancy overall warrants further investigation (75,76,77).

### SCL5A5

The SCL5A5 gene encodes the NIS. NIS is a glycosylated protein with 13 trans-membrane domains and functions to actively transport one I- and two Na+ ions from the sodium ion gradient established by the Na+/K+ ATPase. NIS expression at both the transcriptional level and posttranscriptional level is modulated by the effects of TSH. TSH causes elevated levels of endogenous cAMP in the thyroid cells. These cAMP levels play a role in stimulating several cis-regulatory elements found in the promoter and upstream enhancer sequences on the SCL5A5 gene on chromosome 19 (78,79).

**Figure 6:**
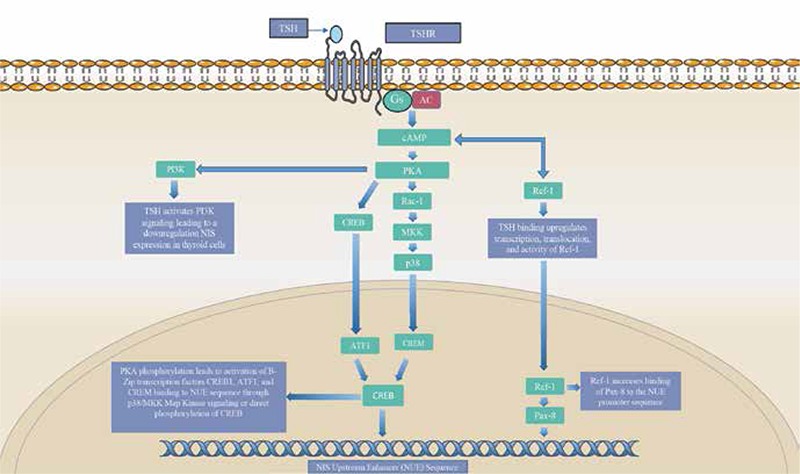
Sodium iodide symporter gene signaling *The sodium Iodide symporter is a transmembrane protein that plays a crucial role in the uptake of iodine by thyroid cells. The SCL5A5 gene is regulated by the activity of TSH on the sodium iodide symporter upstream enhancer sequence (NUE). In this pathway TSH binding to TSHR stimulates an increase in cyclic AMP (cAMP) levels in the cell. Elevated levels of cAMP lead to the activation of protein kinase-A (PKA) dependent p38/MAPK and PKA-cAMP-response element binding protein (CREB) pathways. The p38/MAPK pathway leads to the recruitment of basic-leucine zipper transcription factors ATF1, and cAMP-response modulator to the nucleus. In the nucleus these transcription factors bind to the cAMP-response element binding sites in the NUE and activating the SCL5A5 gene. PKA can also directly phosphorylate the cAMP-CREB to directly regulate cAMP responsive genes such as TSHR, and SCL5A5. Full activation of the SCL5A5 gene is not reached until the PAX-8 transcription factor bindings to the NUE promoter site. TSH signaling upregulates the transcription and nuclear translocation of the oxidative stress factor Ref-1. Ref-1 activates and recruits PAX-8 to bind to the NUE promoter site, leading to full SCL5A5 gene activity. Inhibition of SCL5A5 activity can also occur due to the elevated levels of cAMP. These elevated levels can trigger activation of the PI3K signaling pathway leading to the downregulation of the SLC5A5 gene

The SCL5A5 gene contains an upstream enhancer sequence (NUE) reported to have both a Pax-8, and cAMP-response element (CRE) binding sites. Pax-8 and cAMP-response element modulator (CREM) transcription factors bind to these cis-elements on the upstream enhancer via cAMP-signaling pathways. These elements are stimulated through cAMP activation of protein kinase-A (PKA) dependent pathways in thyroid cells. PKA dependent activation phosphorylates basic-leucine zipper (B-ZIP) proteins, CREM, activating transcription factor-1 (ATF-1), and cAMP-response element binding protein (CREB). Studies have found that the binding of these transcription factors are necessary for the NUE’s cAMP induced activity on thyroidal responsive genes such as TPO, TG, TSHR, and NIS (Figure 6). While CREB binding is important for SLC5A5 transcriptional regulation, full SLC5A regulation is not established without PAX-8 binding. TSH binding increases the expression and translocation of the anti-oxidative stress factor Ref-1. Ref-1 is a nuclear enzyme that reduces Pax-8 and mediates its binding to the NUE sequence. Impaired translocation of this nuclear enzyme has been reported in anaplastic, papillary cell lines as well as in thyroid cancer tissue. TSH also plays a role in the posttranscriptional regulatory role in the transport of NIS to the basolateral membrane (78,79).

While no somatic mutations to NIS have yet been identified, alterations in other genes have been found to be associated with NIS impairment in differentiated thyroid cancers. B-RAFV6000E mutations reduce NIS mRNA expression by inducing the secretion of transforming growth factor-β (TGF-β). TGF-β acts via the SMAD signaling pathway in an MEK/ERK independent pathway. RET/PTC rearrangement impairs the activity of PAX-8 and PKA. Reducing the activity of these two proteins has been demonstrated to reduce recruitment of B-ZIP proteins and reduced binding to the PAX-8 and CRE elements in NUE (79,80,81).

NIS expression has been reported to be reduced in differentiated thyroid carcinomas. One study found reduced NIS mRNA expression in PTC tissue samples when compared to adjacent normal thyroid tissue. NIS expression was also found to be generally reduced in benign thyroid nodules compared with adjacent normal tissue, but to a lesser extent than in PTC tissue samples. It has also been reported that NIS gene expression is impaired in hypo-functioning thyroid tumors when compared to normal thyroid tissue. Quantification of SCL5A5 mRNA levels should be added to the classical prognostic factors currently used to predict the outcomes of patients with differentiated thyroid cancer. SCL5A5 is an excellent candidate for the thyroid cancer mutation panel (82,83,84).

## Conclusion

Thyroid carcinoma is the result of an accumulation of genetic changes that impact its initiation and progression. These genetic alterations, thus, serve as both diagnostic and prognostic markers of thyroid carcinoma. ThyroSeq NGS allows for the use of small amounts of DNA to test for a very broad range of mutations and other genetic alterations, helping identify molecular profiles for the different types of thyroid carcinoma. The genotyping of thyroid samples provides invaluable insight in determining the proper course of treatment when faced with a cytologically indeterminate thyroid nodule.

The genetics of thyroid cancer provides us with a greater understanding of the pathophysiology of the disease than is possible from its cellular morphology. The presence of mutations in the molecular pathology profile can not only help more accurately determine malignancy in the thyroid nodules but help better predict the biology of thyroid carcinoma which will have paramount importance to tailor treatment strategies. The presence of certain genetic alterations may prove indicators of aggressiveness of the cancer and predictors for metastasis. As more studies are conducted, and the genomic profile of thyroid carcinoma is further elucidated, the utility of genomic profiling in the diagnosis and prognostication of thyroid cancer will be solidified.
